# Bilateral abdominal spermatic cord section following peno-scrotal injury

**DOI:** 10.1016/j.eucr.2021.101754

**Published:** 2021-06-15

**Authors:** Pietro Pepe, Paolo Panella, Francesco Savoca, Michele Pennisi

**Affiliations:** Urology Unit - Cannizzaro Hospital, via Messina 829, Catania, Italy

**Keywords:** Spermatic cord section, Trauma of the penis, Scrotal scalp trauma, Testis injury

## Abstract

A 16-year-old man presented to our Emergency hospital for a major traumatic scalp injury of pubic and scrotal region; this occurred as a consequence of an autonomous motorcycle accident. Early surgical esploration showed the bilateral section of spermatic cord in correspondence of the abdominal-pelvic tract; a conservative management was adopted and the patient underwent bilateral intratesticular sperm biopsy for cryopreservation. In the presence of extensive scalp injury of the pubic and scrotum skin a early surgical exploration should be performed and the multidisciplinary decision making should take in account comorbidity, age and impact on the quality of life.

## Introduction

Male genital injuries are rare to occur relatively due to isolated location of the genitals and in most of the cases are caused by road traffic and machinery-related accidents; rarely, the peno-scrotal degloving injury with total amputation of the scrotum has been reported. Although injuries of genitalia are non-lethal, the lesions could be incapacitating and psychologically overwhelming to patients if not treated appropriately.^1^, ^2^, ^3^, ^4^

A case of bilateral spermatic cord section as a consequence of an autonomous motorcycle accident has been reported.

## Case presentation

**Fig. 1:**
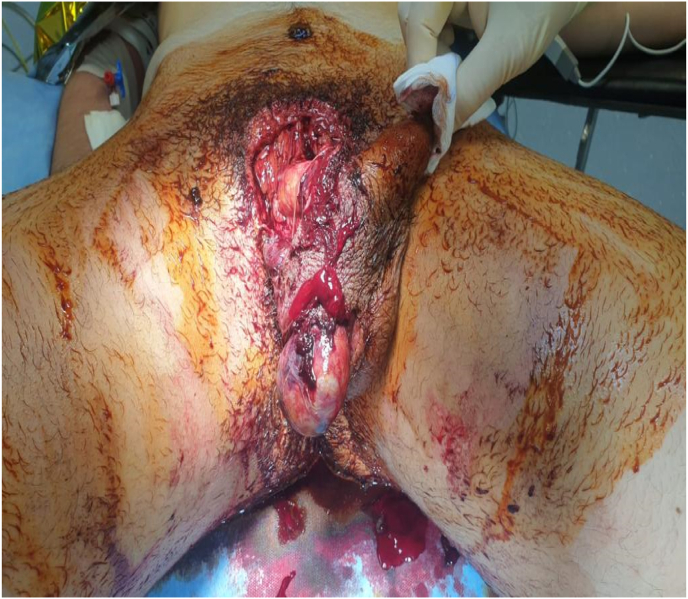
Clinical presentation of peno-scrotal scalp trauma.

**Fig. 2:**
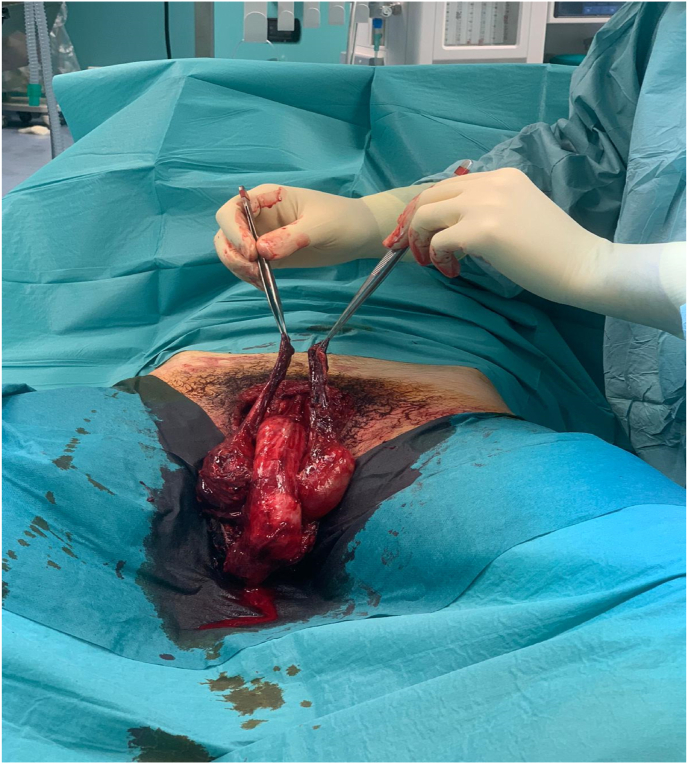
Bilateral abdominal spermatic cord section.

**Fig. 3:**
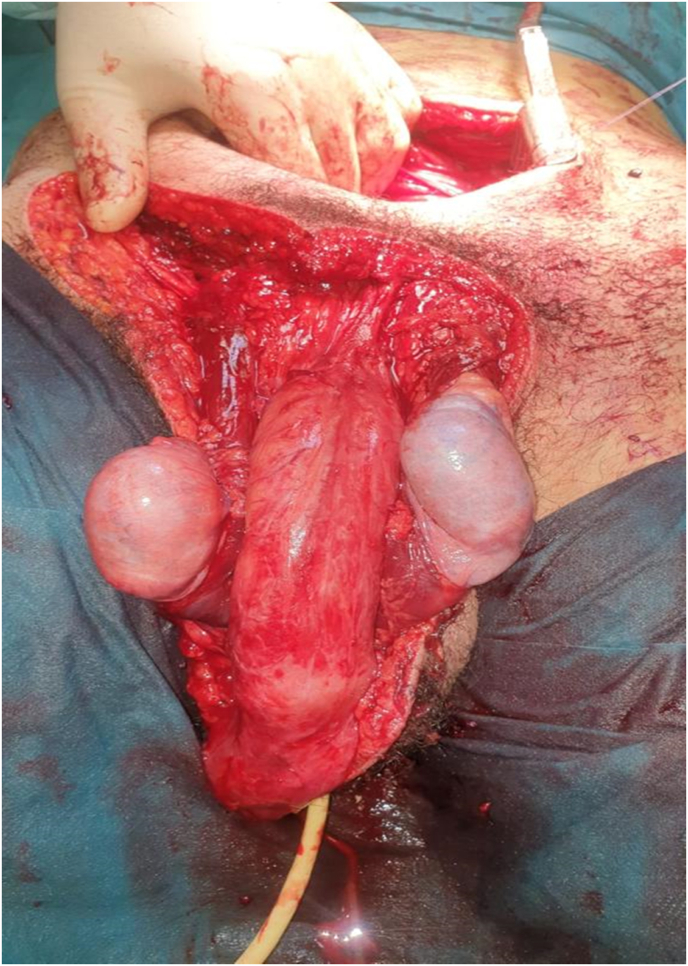
Abdominal surgery approach to detect proximal bilateral spermatic cord; both testes did not showed macroscopic necrotic color (12 hours from the trauma). (For interpretation of the references to color in this figure legend, the reader is referred to the Web version of this article.)

A previously well, 16-year-old man presented after sustaining with a major traumatic injury to pubic and the scrotal region; this occurred as a consequence of an autonomous motorcycle accident during a motocross race occurred about 12 hours before hospital admission. Physical examination revealed extensive scalp injury of the pubic and scrotum skin involving bilateral testis and penis (Fig. 1); the patient underwent emergency surgery for evacuation of a haematoma and debridement of necrotic tissue. Preoperatively brain, lung and abdominal computed tomography (CT) scan did not showed bleeding internal lesions. The emergency colordoppler ultrasound (CDU) of the testis demonstrated the bilateral integrity of the tunica albuginea; in addition, an apparent normal flow of intratesticular vessel was recordered.^5^ Surgical esploration showed the testes anchored to the gubernaculum testis and the bilateral section of spermatic cord (Fig. 2) in correspondence of the abdominal-pelvic tract, therefore abdominal incision was performed to search the proximal spermatic cord tracts those presented a full section without active bleeding. A vascular anastomosis between the dissected spermatic cord was not recommended by vascular surgeon therefore a suture to prevent bleeding was performed. Although the bilateral spermatic cord section both testes did not showed the typical necrotic color (Fig. 3) therefore hoping that cremasteric and deferencial vessels could spray the testicles a conservative management was adopted. The next morning the patient underwent bilateral intratesticular sperm biopsy for cryopreservation and a total of 14 spermatozoa were picked up and deposited at the assisted reproduction center. After 1 week the testosterone level was equal to 1 ng/ml therefore hormonal substitution therapy was given; in addition, CDU of the testis did not showed intraparenchimal blood flow that was confirmed by microbubble contrast agent Sonovue administration. The patient was discarged 20 days from trauma in good general conditions without infections complications; the patient still today is assisted by the psychologist and at 3 months follow up he refer a normal erectile function.

## Discussion

Genitourinary injury is present in approximately 10% of cases of abdominal trauma^1^ and about 65% involve the external genitalia.^2^^,^^3^ There are two broad domains to consider when evaluating scrotal trauma: blunt (75% of the cases) vs. penetrating injury. This increased incidence in males is secondary to both anatomical considerations and increased participation in activities such as contact sports, violent interaction, and war activities.^4^ Avulsion injuries resulting in skin loss are typically a result of rapid deceleration mechanisms; the management of penoscrotal avulsion includes a thorough cleaning and debridement of devitalized tissues and the exposed tissues are covered with viable flaps from the remaining skin.

In our case, the first to our knowledge reported in literature, an extensive scalp injury of the pubic and scrotum skin involving bilateral testis and penis only the bilateral spermatic cord section was reported. Although CDU^5^ showed an apparent normal flow of intratesticular testicular vessels with the integrity of tonaca albuginea only the surgical exploration allowed to demonstrate the severity of the trauma secondary to abdominal bilateral spermatic cord section. In very rarely traumatic cases surgical options should be undertaken in multidisciplinary team to improve the quality of life of the patient; in our case, in consideration of the young age, the patient underwent an early bilateral intratesticular sperm biopsy for cryopreservation following the trauma repair.

## Conclusion

In the presence of extensive scalp injury of the scrotum skin involving bilateral testis and penis an early surgical exploration should be mandatory; in the presence of isolated bilateral spermatic cord section the multidisciplinary decision making should take in account comorbidity, age and impact on the quality of life for the patient.

## Ethical approval and consent to participate

All procedures performed in this study involving human participants were in accordance with the ethical standards of the institutional and/or national research committee and with the 1964 Helsinki Declaration and its later amendments or comparable ethical standards.

## Informed consent

Written informed consent was obtained from the patient for his anonymized information to be published in this article.

## Declaration of competing interest

The authors declare that they have no conflicts of interest.

This research did not receive any specific grant from funding agencies in the public, commercial, or not-for-profit sectors.
